# *SKIL*/SnoN attenuates TGF-β1/SMAD signaling-dependent collagen synthesis in hepatic fibrosis

**DOI:** 10.17305/bb.2023.9000

**Published:** 2023-12-01

**Authors:** Cheng Chi, Xifeng Liang, Tianyu Cui, Xiao Gao, Ruixia Liu, Chenghong Yin

**Affiliations:** 1 Central Laboratory, Beijing Obstetrics and Gynecology Hospital, Capital Medical University. Beijing Maternal and Child Health Care Hospital, Beijing, China; 2 School of Nursing, Jining Medical University, Jining, Shandong, China; 3 School of Nursing, Weifang Medical University, Weifang, Shandong, China

**Keywords:** Ski-related novel protein N (SnoN), SKIL, transforming growth factor-β1 (TGF-β1), hepatic fibrosis (HF), bioinformatics

## Abstract

The ski-related novel protein N (SnoN), encoded by the *SKIL* gene, has been shown to negatively regulate transforming growth factor-β1 (TGF-β1) signaling pathway. However, the roles of SnoN in hepatic stellate cell (HSC) activation and hepatic fibrosis (HF) are still unclear. To evaluate the role of SnoN in HF, we combined bulk RNA sequencing analysis and single-cell RNA sequencing analysis to analyze patients with HF. The role of *SKIL*/SnoN was verified using liver samples from rat model transfected HSC-T6 and LX-2 cell lines. Immunohistochemistry, immunofluorescence, PCR, and western blotting techniques were used to demonstrate the expression of SnoN and its regulatory effects on TGF-β1 signaling in fibrotic liver tissues and cells. Furthermore, we constructed a competitive endogenous RNA regulatory network and potential drug network associated with the *SKIL* gene. We identified the *SKIL* gene as a differentially expressed gene in HF. SnoN protein was found to be widely expressed in the cytoplasm of normal hepatic tissues, whereas it was almost absent in HF tissues. In the rat group subjected to bile duct ligation (BDL), SnoN protein expression decreased, while TGF-β1, collagen III, tissue inhibitor of metalloproteinase 1 (TIMP-1), and fibronectin levels increased. We observed the interaction of SnoN with p-SMAD2 and p-SMAD3 in the cytoplasm. Following SnoN overexpression, apoptosis of HSCs was promoted, and the expression of HF-associated proteins, including collagen I, collagen III, and TIMP-1, was reduced. Conversely, downregulation of SnoN inhibited HSC apoptosis, increased collagen III and TIMP-1 levels, and decreased matrix metalloproteinase 13 (MMP-13) expression. In conclusion, SnoN expression is downregulated in fibrotic livers and could attenuate TGF-β1/SMADs signaling-dependent de-repression of collagen synthesis.

## Introduction

Hepatic fibrosis (HF) is a complex pathophysiological process characterized by the replacement of normal liver tissue by scar tissue, resulting from the dysregulation of scar formation in the liver [1]. Hepatocyte cell death, hepatic stellate cells (HSCs) activation, and inflammation are crucial incidences of HF [2]. A particular hallmark of HF is the uncontrolled activation of HSCs, which transform into myofibroblasts, resulting in excessive deposition of extracellular matrix (ECM) dominated by collagen [3, 4]. HF generally occurs in patients with hepatic dysfunction which is associated with various signaling pathways [5]. Among these factors, transforming growth factor-β1 (TGF-β1) induces autophosphorylation of TGF-β1 receptors, resulting in the activation of receptor-mediated SMADs (R-SMADs). This activation leads to the release of p-SMAD2/3 from the SMAD anchor, enabling them to form a complex with Co-SMAD4 and subsequent translocation to the nucleus. As a consequence, the expression of a variety of fibrocollagen genes in HSCs is increased, including collagen I, collagen III, fibronectin (FN), etc. [6]. Therefore, TGF-β1 is the most potent profibrogenic cytokine, that plays a key role in HSC activation and ECM deposition [3, 4].

The ski-related novel protein N (SnoN) is a variant of the Ski-Sno superfamily and is encoded by the *SKIL* gene [7]. As an inhibitory factor of TGF-β1 signaling, SnoN protein can block TGF-β1 signaling pathway in both the cytoplasm and nucleus [7]. SnoN has been shown to negatively regulate TGF-β1 signaling pathway by interacting with p-SMADs and the co-mediator SMAD4 (Co-SMAD4) in tumor cells and renal tubular epithelial cells [8]. Although there is increasing evidence indicating that SnoN protein plays an important role in driving fibrotic responses, including in tumor, kidney, heart, and lung tissues [9–11], the role of SnoN in HF has not yet been clarified. Since the expression and function of SnoN differ in different tissues and diseases, it is important to investigate the role of SnoN in liver tissue and HF. Our previous study showed that a traditional Chinese herbal medicine attenuates the process of HF by increasing SnoN protein expression and inhibiting the TGF-β1/SMAD signaling pathway [12]. Thus, we hypothesized that SnoN plays an important role in the formation of HF via binding to SMAD proteins and regulating the TGF-β1/SMAD signaling pathway.

In this study, we employ a series of bioinformatics approaches to point out the key pathogenic genes between HF affected liver tissue samples and normal liver tissue samples and explore the potential biological functions of *SKIL* as a characteristic gene. The aim of this study was to demonstrate the role of *SKIL/*SnoN in the fibrosis process and provide evidence on its regulatory effect in HF.

## Materials and methods

### Bulk RNA sequencing analysis

Datasets from multiple researches were used for integrated analysis in this study, which were downloaded from the Gene Expression Omnibus (GEO) database (https://www.ncbi.nlm.nih.gov/). Bulk RNA-seq data were obtained from datasets GSE139602 and GSE84044. Differential expression analysis was performed by R package limma. Differentially expressed genes (DEGs) were selected under a criterion of |log2 Fold Change (FC)| > 1 and *P* value < 0.05. Gene Ontology (GO), Kyoto Encyclopedia of Genes and Genomes (KEGG) pathway, and Disease Ontology (DO) enrichment analyses were performed with the R packages: clusterProfler and DOSE. HF patients in the GSE84044 dataset were divided into high expression group and low expression group according to the median value of *SKIL*. Gene set variation analysis (GSVA) was conducted by GSVA package to further validate different biological procedures between the two groups [13].

### Single-cell RNA sequencing analysis

Single-cell transcriptomic profiles of five cirrhotic and five normal human liver tissues were obtained from GSE136103. The data were analyzed via the R package Seurat [14] and normalized using the SCTransform method. We identified the top 2000 variable genes and applied dimensions reduction using the uniform manifold approximation and projection (UMAP) method. The cells distribution was annotated by the CellMarker database (http://bio-bigdata.hrbmu.edu.cn/CellMarker/index.html).

### Animal experiments

A total of 90 male Wistar rats in specific pathogen-free (SPF) grade, weighting 160–200 g, were purchased from Vital River Laboratory Animal Technology Company (Beijing, China). Fifty rats were used for the liver fibrosis model induced by common bile duct ligation (BDL). The rats were fed adaptively for one week and then randomly divided into two groups: the control group (*n* ═ 10) and the BDL group (*n* ═ 40). BDL operations were performed as previously described [15, 16]. The BDL group was further assigned into four subgroups: BDL 7d, 14d, 28d, and 42d (10 rats in each subgroup), in which the rats were sacrificed at the 7th, 14th, 28th, and 42nd postoperative day, respectively. The rats in the control group were sacrificed at the 42nd postoperative day. Forty rats were used for carbon tetrachloride (CCL4)-induced liver fibrosis model. The rats were fed adaptively for one week and then randomly assigned into two groups: the control group (*n* ═ 8) and the CCL4 group (*n* ═ 32). The CCL4 group was further divided into four groups, and the rats were sacrificed at 1 week (*n* ═ 8), 3 weeks (*n* ═ 8), 5 weeks (*n* ═ 8), and 7 weeks (*n* ═ 8), respectively. The rats of the control group were sacrificed at 7 weeks. The rats in the CCL4 group were subcutaneously injected with 60% CCL4 olive oil solution, while the rats in the control group were subcutaneously injected with normal saline. Liver and blood samples were collected from 90 rats, then snap-frozen and stored in liquid nitrogen until being assayed.

### Liver biochemistry indices

Blood samples were centrifuged at 4000 × *g* for 5 min to separate the serum from other blood components. Then the serum samples were tested to measure the levels of aspartate amino-transaminase (AST), alanine amino-transaminase (ALT), total bilirubin (TBIL), and direct bilirubin (DBIL) using an automatic biochemistry analyzer (Abbott Laboratories, Chicago, IL, USA), according to the manufacturer’s protocol.

### Histology and immunohistochemistry

Haematoxylin and eosin (HE) staining, Masson staining, and immunohistochemistry were performed. The following primary antibodies were used in immunohistochemistry: polyclonal rabbit anti-rat SnoN (1:200 dilution; Abcam, Cambridge, UK), collagen III (1:50 dilution, Abcam), TGF-β1 (1:50 dilution; Santa Cruz, OR, USA), tissue inhibitor of metalloproteinase 1 (TIMP-1) (1:50 dilution, Santa Cruz), and matrix metalloproteinase 13 (MMP-13) (1:50 dilution, Santa Cruz).

### Cell cultures

HSC line HSC-T6 and Lieming Xu-2 (LX-2) cell lines were purchased from Cancer Institute of Chinese Academy of Medical Sciences and Procell Life Science and Technology Co., Ltd, respectively. HSC-T6 cells were cultured on plates in Dulbecco’s modified Eagle’s medium (DMEM, HyClone, UT, USA), supplemented with 10% fetal bovine serum (FBS; Gibco, MA, USA). LX-2 cells were cultured in specific medium containing DMEM, 10% FBS, and 1% P/S. The cells were maintained at an atmosphere of 5% CO_2_ and 37 ^∘^C.

### Vector and transfection

The SnoN coding sequence was retrieved from GenBank (NM_005414.4), synthesized and inserted into the GV287 vector. This vector was digested using BamHI/AgeI. Lentiviral packaging was carried out by Genechem (Shanghai, China). Before transfection the medium of the cells was replaced by serum- and antibiotic-free DMEM. The lentiviruses transfection reagent (Genechem) was diluted by serum and antibiotic-free medium based on multiplicity of infection (MOI) and cell counts. HSC-T6 cells were grouped as follows: control group, Ad-EGFP group, Ad-SnoN group, si-FITC group, and si-SnoN group. The Ad-EGFP group was transfected with empty lentiviral vector which can express enhanced green fluorescent protein (EGFP); the Ad-SnoN group was transfected with SnoN gene lentiviral vector; the si-FITC group was transfected with fluorescein isothiocyanate (FITC) labeled siRNA; the si-SnoN group was transfected with siRNA targeting SnoN RNA.

**Table 1:** PCR primer sequences

**Gene**	**Forward sequence**	**Reverse sequence**
SnoN	5’-CCATTCAATGCCCCATCCT-3’	5’-AGTTCGTGGCCGCAATAAAG-3’
*TGFβ1*	5’-ATACGCCTGAGTGGCTGTCT-3’	5’-TGGGACTGATCCCATTGATT-3’
*SMAD2*	5’-TGAGCTTGAGAAAGCCATCA-3’	5’-TGTGTCCCACTGATCTACCG-3’
*SMAD3*	5’-CATTACCATCCCCAGGTCAC-3’	5’-CGTAACTCATGGTGGCTGTG-3’
*TIMP1*	5’-TCCTCTTGTTGCTATCATTGATAGCTT-3’	5’-CGCTGGTATAAGGTGGTCTCGAT-3’
*MMP13*	5’-GGAAGACCCTCTTCTTCTCA-3’	5’-TCATAGACAGCATCTACTTTGTC-3’
*COL1A1*	5’-CATGTTCAGCTTTGTGGACCT-3’	5’-GCAGCTGACTTCAGGGATGT-3’
*COL3A1*	5’-GGGATCCAATGAGGGAGAAT-3’	5’-GCTCCATTCACCAGTGTGTTT-3’
*GAPDH*	5’-CCTGCCAAGTATGATGACATCAAGA-3’	5’-GTAGCCCAGGATGCCCTTTAGT-3’

SnoN: Ski-related novel protein N; TGF-β1: Transforming growth factor beta 1; SMAD: Suppressor of mothers against decapentaplegic protein; TIMP-1: Tissue inhibitor of metalloproteinase 1; MMP13: Matrix metalloproteinase 13; COL: Collagen; GAPDH: Glyceraldehyde-3-phosphate dehydrogenase.

The pcDNA 3.1 vector was digested by exonuclease III (Exo III) to construct a vector overexpressing SnoN. LX-2 cells were divided into the OE-SnoN group, the TGF-β1 group, and the OE-SnoN + TGF-β1 group. The OE-SnoN group was transfected with SnoN-expressing vector, the TGF-β1 group was stimulated with 5 ng/mL TGF-β1, and the OE-SnoN + TGF-β1 group was stimulated with 5 ng/mL TGF-β1 after transfection with SnoN-expressing vector.

### Immunofluorescence

HSC-T6 cells were cultured on 10 cm dishes and stimulated for 48 h in absence or presence of 5 ng/mL rTGF-β1 (Sigma, MO, USA). The following primary antibodies were used: polyclonal rabbit anti-rat SnoN (1:200 dilution, Abcam), p-SMAD2 (1:100 dilution, Cell Signaling Technology, MA, USA), p-SMAD3 (1:100 dilution, Cell Signaling Technology), secondary antibody (1:100 dilution, Life Technology, CA, USA), FITC-tagged secondary antibody (1:100 dilution, Life Technology).

### Co-immunoprecipitation

Co-immunoprecipitation (Co-IP) cells were stimulated by rTGF-β1 (5 ng/mL) for 18 h and then lysed in cell lysis buffer supplemented with Halt Protease and Phosphatase Inhibitor Cocktail (Thermo Fisher Scientific, NY, USA). Co-IP was performed according to the Pierce Co-Immunoprecipitation Kit (Thermo Fisher Scientific).

### Western blotting

Liver samples and cells were lysed with the radio-immunoprecipitation assay (RIPA) buffer (Thermo Fisher Scientific) at 4 ^∘^C. Total proteins were separated using 10% sodium dodecyl sulfate-polyacrylamide gels and then transferred to polyvinylidene fluoride membranes (Merck Millipore, MA, USA). The membranes were incubated with primary antibodies, including TGF-β1 (dilution 1:200; Abcam), p-SMAD2 (dilution 1:300; Cell Signaling Technology), p-SMAD3 (dilution 1:300; Cell Signaling Technology), SnoN (dilution 1:400; Santa Cruz; sc-136958), collagen III (dilution 1:100; Abcam), FN (dilution 1:500; Santa Cruz; sc-8422), TIMP-1 (dilution 1:200; Santa Cruz; sc-21734), and MMP-13 (dilution 1:100; Santa Cruz; sc-515284) overnight at 4 ^∘^C. Beta actin (β-actin) was used as a loading control using a mouse monoclonal antibody (dilution 1:400; Abcam). The peroxidase-conjugated anti-rabbit or anti-mouse secondary antibodies (dilution 1:10,000; Santa Cruz) were used. Bound antibodies were visualized using the enhanced chemiluminescence (ECL) western blotting detection reagents (Merck Millipore) and analyzed with Image Lab software.

### Quantitative real-time polymerase chain reaction (qRT-PCR)

The total RNA was extracted from liver samples with Trizol (Invitrogen, MA, USA) and was reverse transcribed (Fermentas, ON, CA). The resultant cDNA was subjected to qRT-PCR, and primer sequences for SnoN, TGF-β1, SMAD2, SMAD3, collagen I, collagen III, TIMP-1, MMP-13, and housekeeping gene glyceraldehyde-3-phosphate dehydrogenase (*GAPDH*) were shown in Table 1. qRT-PCR was performed with PowerSYBR Green PCR Master Mix (Applied Biosystems, CA, USA) using a 7500 real-time PCR instrument (Applied Biosystems). The PCR protocol was as follows: denaturation at 95 ^∘^C for 5 min, then 95 ^∘^C for 15 s, and 60 ^∘^C for 1 min for 40 cycles. The amount of *GAPDH* cDNA was used to normalize the sample amounts used for determinations.

### Immune infiltration analysis

The relative proportion of immune cells in HF was quantified by calculating the immune cell infiltration by CIBERSORT. The 22 types of invasive immune cells were analyzed and visualized using the R package corrplot.

### Correlation between characteristic genes and infiltrating immune cells

We used Spearman’s rank correlation analysis in R to investigate the correlation between the characteristic gene and infiltrating immune cell levels. We visualized the identified associations using the chart technique with the ggplot2 package.

### Construction of competitive endogenous RNA and potential drugs network

In order to construct the competitive endogenous RNA (ceRNA) regulatory network of *SKIL* genes, we first used the GSE74872 dataset and the GSE134146 dataset to screen differentially expressed miRNAs (DE-miRNAs) and circRNAs (DE-circRNAs), (|log2 FC| > 1 and *P* value < 0.05). Then the targetscan database (http://www.targetscan.org/) was used to predict miRNA targeting *SKIL* gene, and the starbase database (https://starbase.sysu.edu.cn/) was used to predict circRNA targeting corresponding miRNA. Finally, we took miRNA–mRNA relationship pairs and miRNA–circRNA relationship pairs with opposite expression trends. The ceRNA regulatory networks were constructed and visualized using the Cytoscape software [17]. We used the CTD database (http://ctdbase.org/) to predict the potential drugs targeting the *SKIL* gene and construct a gene-chemical compound network using the Cytoscape software [18].

### Ethical statement

The animal experimental methods used in this study were approved by the Ethics Committees of Beijing Friendship Hospital of Capital Medical University (Beijing, China) and Jining Medical University (jnmc-2019-zr-004). All animal experiments were conducted in accordance with the National Institutes of Health Guide for the Care and Use of Laboratory Animals (NIH Publication no. 8023, revised 1978).

### Statistical analysis

All statistical analyses were performed using SPSS software version 20.0 (IBM, NY, USA). The data consistent with normal distribution was described by mean ± standard deviation. One-way ANOVA was used for the comparisons among independent samples of multiple groups. For all tests, a *P* value < 0.05 was considered to be statistically significant. We used the ARRIVE1 checklist when writing our report.

## Results

### Bulk RNA sequencing and single-cell RNA sequencing analysis

**Figure 1.**
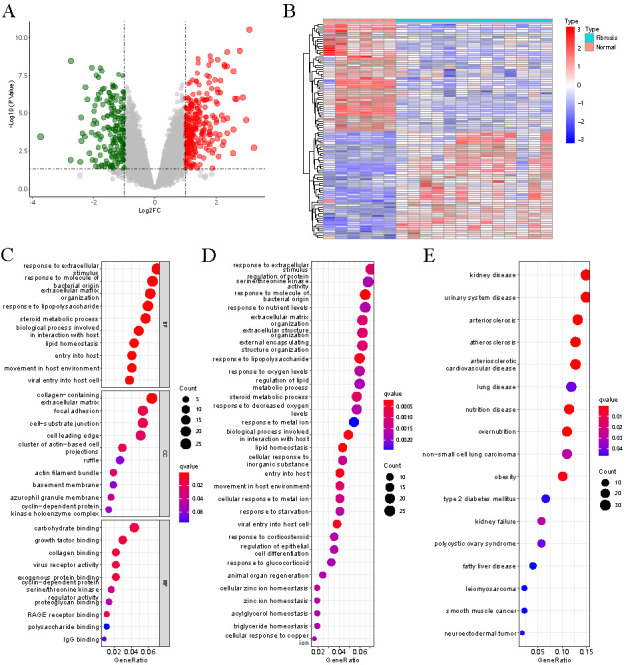
**Hepatic fibrosis-associated DEGs and their enrichment analysis.** (A) Volcano map of DEGs: The red dot indicates the upregulated gene expression, the blue dot indicates the downregulated gene expression, and the gray dot indicates that there was no significant difference between the expression of these genes; (B) Heat map of top 100 DEGs: Each small square indicates each gene, and its color indicates the expression level of the gene. The greater the expression difference, the darker the color (red indicates high expression and blue indicates low expression); (C) GO analysis; (D) KEGG analysis; (E) DO analysis. DEGs: Differentially expressed genes; GO: Gene Ontology; KEGG: Kyoto Encyclopedia of Genes and Genomes; DO: Disease Ontology; RAGE: Receptor for advanced glycation endproducts.

The DEGs in HF were identified by analyzing the sample data of five early fibrotic liver tissue samples and six normal tissue samples in the GSE139602 dataset. We identified a total of 389 DEGs, including 151 upregulated genes and 238 downregulated genes (Figure 1A), among which *SnoN* was downregulated (|logFC| > 1 and *P* < 0.05). Top 100 DEGs were displayed in a heatmap (Figure 1B).

**Figure 2.**
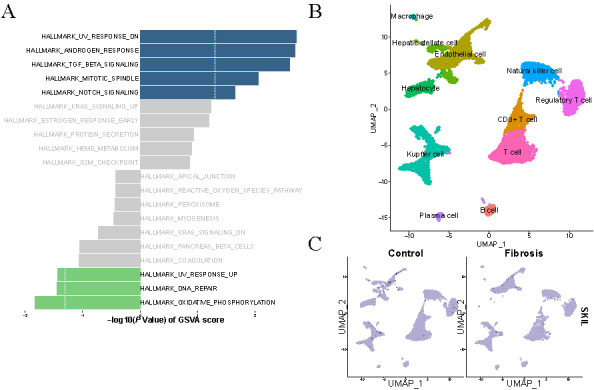
**Cluster analysis by the gene set variation analysis (GSVA) and the uniform manifold approximation and projection (UMAP).** (A) Biological processes and pathways associated with fibrotic liver tissue sample genes in the GSE84044 dataset. Hallmark section of GSVA; (B) Clusters annotation and cell types identification via UMAP; (C) The expression level of SnoN in HF and control groups. SnoN: Ski-related novel protein N; HF: Hepatic fibrosis; TGF-β1: Transforming growth factor beta 1.

To understand the biological functions of HF-related DEGs, we performed the DO, KEGG, and GO enrichment analysis for DEGs. GO enrichment analysis involved three entries: biological processes (BP), cellular components (CC), and molecular functions (MF) [19]. A total of 176 GO entries (including 161 BP entries, 7 CC entries, and 8 MF entries), 175 KEGG pathway, and 17 DO entries were enriched. The results of BP analyses showed that DEGs were remarkably involved in response to extracellular stimulus, in response to molecules of bacterial origin and in response to ECM organization (Figure 1C). CC analysis suggested that collagen-containing ECM, focal adhesion, and cell-substrate junction were mainly enriched (Figure 1C). MF analysis indicated that DEGs were majorly located in carbohydrate binding, growth factor binding, and collagen binding (Figure 1C). In the KEGG pathways, the results indicated that DEGs were mainly involved in response to extracellular stimulus, molecule of bacterial origin, and in response to nutrient levels (Figure 1D). The enriched DO was mainly related to kidney disease, urinary system disease, arteriosclerosis, and arteriosclerotic cardiovascular disease (Figure 1E). To explore the discrepancies in signaling pathways between the high- and low-risk groups, fibrotic liver tissue samples from the GSE84044 dataset were used for GSVA enrichment analysis. The potential mechanism of *SKIL* gene related to HF was analyzed at the Hallmark level. The results showed that *SKIL* genes were mainly enriched in UV-response-DN, Androgen-response, and TGF-β1 signaling pathways (Figure 2A).

Through scRNA and UMAP analysis, we identified a total of 11 cell types, including macrophages, HSCs, endothelial cells, hepatocyte, natural killer cells, regulatory T cells, CD8+ T cells, T cells, Kupffer cells, B cells, and plasma cells (Figure 2B). It was notable that the expression level of SnoN in HSCs, hepatocyte, and endothelial cells was higher in the fibrotic tissue than the normal liver tissue (Figure 2C).

### Liver injury in bile duct ligation (BDL) model

**Figure 3.**
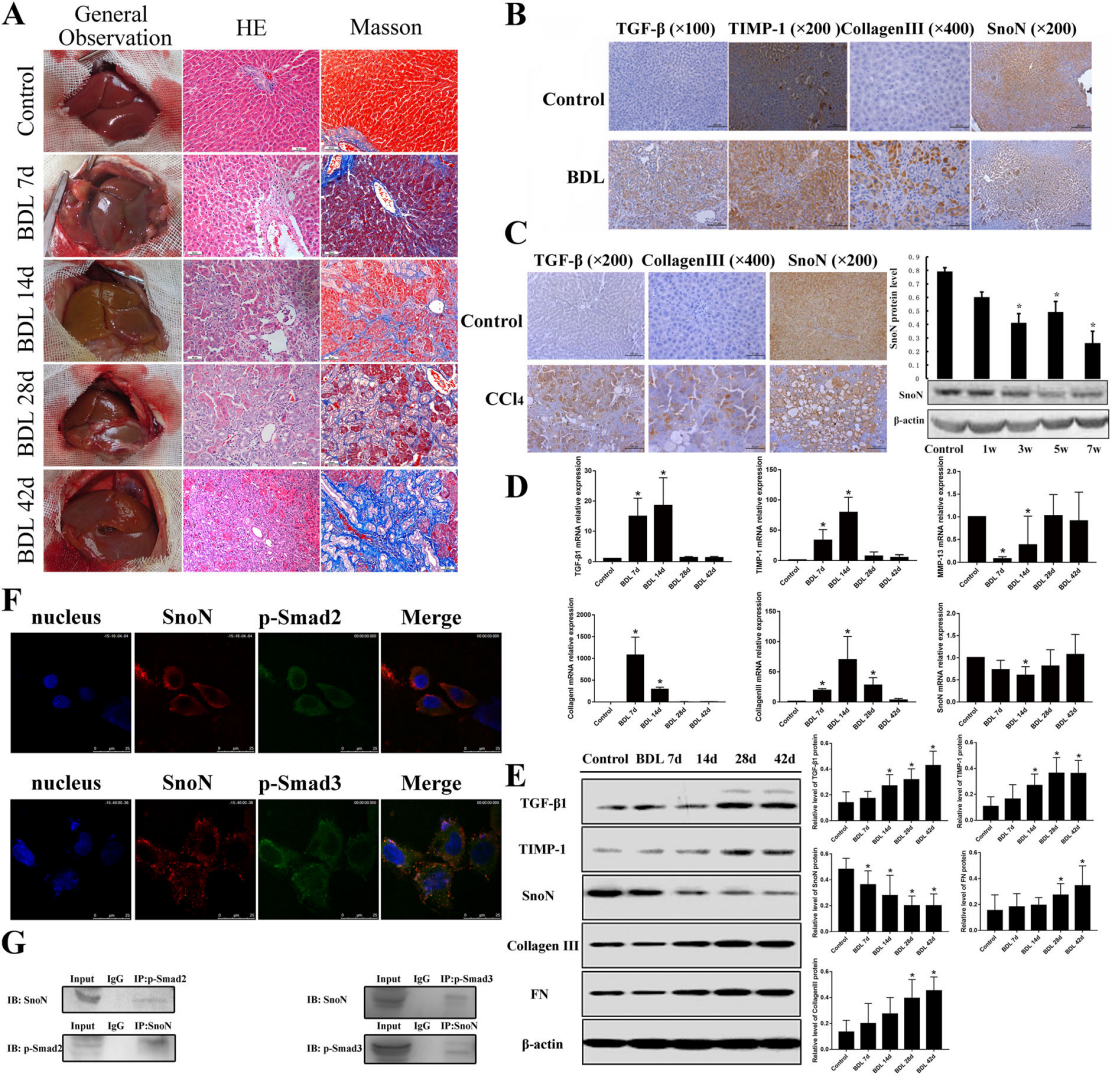
**SnoN regulates fibrosis-associated proteins through its interaction with p-SMAD2 and p-SMAD3**. (A) Gross specimen morphology for the BDL and control groups. Histologic examination of liver sections was performed using hematoxylin and eosin and Masson’s trichrome staining (magnification × 200); (B) The positive expression location of TGF-β1, TIMP-1, collagen III, and SnoN analyzed by immunohistochemistry assay (magnification × 200); (C) The expressions of TGF-β1, collagen III, and SnoN protein in liver tissue of rats in each group were observed by immunohistochemistry, the relative protein expression of SnoN in liver tissue of rats in each group was detected by western blot. * *P* < 0.05 compared with the control group; (D) Quantitative PCR analysis of the mRNA expression of TGF-β1, TIMP-1, collagen III, collagen I, MMP-13, and SnoN; (E) As time went on, the expression of TGF-β1, TIMP-1, collagen III, and FN proteins increased, while SnoN protein decreased. Data are expressed as mean ± SD, *n* ═ 10 rats/group. * *P* < 0.05 compared with the control group; (F) Immunofluorescent staining showed the co-location of SnoN and p-SMAD2, the co-location of SnoN and p-SMAD3; (G) The interaction of SnoN and p-SMAD2 and the interaction of SnoN and p-SMAD3 in HSC-T6 cells were examined by co-IP, followed by immunoblot analysis. IgG represents a control antibody used for IPs. BDL: Bile duct ligation; HE: Hematoxylin and eosin; TGF-β1: Transforming growth factor beta 1; TIMP-1: Tissue inhibitor of metalloproteinase 1; SnoN: Ski-related novel protein N; MMP-13: Matrix metalloproteinase 13; FN: Fibronectin; SMAD: Suppressor of mothers against decapentaplegic protein; HSC: Hepatic stellate cells; IP: Immunoprecipitation; co-IP: Co-immunoprecipitation.

The livers of the rats from the control group showed a soft, ruddy, shiny, smooth, delicate surface with a clear edge (Figure 3A). In contrast, the livers from the BDL groups turned to be yellow dyed, shrunken, the coverings were adherent, blunt edge appeared, and with an uneven surface.

We analyzed the histological changes of the liver tissues by HE and Masson staining. In the control group, the liver tissues exhibited normal lobular architecture with central veins, radiating hepatic cords, and no regenerated collagen fibers (Figure 3A). In the BDL groups, membrane-like intervals of collagen fibers, fibroblasts, and small capillaries were formed, and the adjacent cells were connected with the adjacent hyperplasia, resulting in the formation of pseudo lobules.

BDL surgery also significantly increased the serum levels of ALT, AST, TBIL, and DBIL compared with the control group (all *P* < 0.01, Table 2), suggesting that the BDL surgery induced severe damage to liver function.

**Table 2:** Serum biochemical indicators of liver function

**Group**	**TBIL (µmol/L)**	**DBIL (µmol/L)**	**ALT (U/L)**	**AST (U/L)**
Control	0.89±0.23	0.48±0.44	35.50±3.02	120.50±15.29
BDL 7d	80.81±61.94^*^	74.09±58.16^*^	75.67±33.07^*^	300.50±175.63^*^
BDL 14d	117.58±33.28^*^	107.54±29.90^*^	86.67±18.99^*^	326.17±60.18^*^
BDL 28d	82.44±39.55^*^	74.64±36.08^*^	96.67±39.99^*^	397.67±190.00^*^
BDL 42d	103.46±24.58^*^	93.80±19.88^*^	93.00±27.30^*^	313.00±65.48^*^

Values are shown as mean ± SD. * *P* < 0.05 vs control group. BDL: Bile duct ligation; TBIL: Total bilirubin; DBIL: Direct bilirubin; ALT: Alanine aminotransferase; AST: Aspartate aminotransferase.

### Effects of BDL surgery on SnoN and TGF-**β**1 signaling-associated genes and proteins

Positive immunostaining of TGF-β1, TIMP-1, collagen III, and SnoN were observed predominantly in the cytoplasm (Figure 3B). Compared with normal tissue, the expression of TGF-β1 and collagen III in liver tissue of rats injected with CCL4 increased significantly at seven weeks, and the expression of SnoN protein decreased significantly at three weeks and reached the minimum at seven weeks (*P* < 0.05, Figure 3C). Compared with the control group, in the BDL surgery group, there was a significant increase in the mRNA levels of TGF-β1, TIMP-1, collagen I, and collagen III at 7d and 14d (*P* < 0.05), while there was no significant difference later at 28d and 42d (*P* > 0.05), except for collagen III at 28d (*P* < 0.05, Figure 3D). On the contrary, gene expression of SnoN and MMP-13 mRNA in BDL groups showed a tendency to decrease first and then return back to normal levels. It was notable that the BDL 14d group showed a significant decrease in mRNA levels of SnoN and MMP-13, compared with the control group (*P* < 0.05, Figure 3E). The expression of TGF-β1 and TIMP-1 proteins increased significantly in BDL 14d, 28d, and 42d groups, compared with the control group (Figure 3E). The expression of collagen III and FN proteins increased significantly in BDL 28d and 42d groups, compared with the control group (Figure 3E). On the contrary, the expression of SnoN protein decreased with time (Figure 3E).

### Co-localization and interaction between SnoN and p-SMAD2/3 proteins

We found that SnoN, p-SMAD2, and p-SMAD3 were all localized in the cytoplasm (Figure 3F and 3G). The results of immunofluorescence revealed that the co-localization of SnoN and p-SMAD2, as well as the co-localization of SnoN and p-SMAD3 expression were detected in the cytoplasm of HSC-T6 cells (Figure 3F). In addition, the interactions between SnoN and p-SMAD2 as well as SnoN and p-SMAD3 were observed by co-IP (Figure 3G).

### Transfection efficiency and the expression of SnoN in transfected HSC-T6 cells

Observed by inverted fluorescence microscope, the green fluorescent protein (GFP) expressed in Ad-EGFP groups, and emitted green fluorescence was stimulated by ultraviolet light. We counted the number of cells under fluorescent and natural light, and then calculated the transfection efficiency of lentiviruses by the ratio. The infection efficiency of lentiviruses increased within 72 h. The infection efficiency of lentiviruses was 20.58%, 50.69%, and 93.56%, when the MOI was 10, 20, and 50 at 72 h. As HSC-T6 cells die mostly when MOI was larger than 50, we adopted the value of 50 as MOI and 72 h as the intervention time.

**Figure 4.**
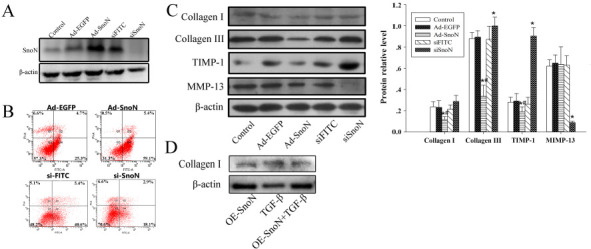
**Effect of SnoN on apoptosis and fibrosis-associated proteins**. (A) The expression of SnoN protein after transfection. *P < 0.05 compared with the control group; (B) Apoptosis of cells in each group was detected by flow cytometry with Annexin V-FITC/PI labeling. *P < 0.05 compared with the control group; (C) Western blot analysis of protein expression of collagen I, collagen III, TIMP-1, and MMP-13 after transfection. *P < 0.05 compared with the control group; ^#^*P* < 0.05 compared with the Ad-EGFP group; ^%^*P* < 0.05 compared with the si-FITC group; (D) Western blot analysis of protein expression of collagen I. SnoN: Ski-related novel protein N; EGFP: Enhanced green fluorescent protein; FITC: Fluorescein isothiocyanate; PI: Propidium iodide; TIMP-1: Tissue inhibitor of metalloproteinase 1; MMP-13: Matrix metalloproteinase-13; TGF-β1: Transforming growth factor beta 1.

We analyzed the expression of SnoN protein in each group by western blotting (Figure 4A). The expression of SnoN protein was increased significantly in Ad-SnoN group, compared with the control group and Ad-EGFP group (both *P* < 0.05). Notably, the expression of SnoN protein was decreased significantly in si-SnoN group, compared with the control group and si-FITC group (both *P* < 0.05).

### Effect of SnoN on apoptosis and fibrosis-associated proteins

Annexin V-FITC/propidium iodide (PI) combined labeling flow cytometry was used to detect the apoptosis of HSC-T6 cells in each group, in which the Q4 region represented early apoptotic cells, and the percentage of total cells in that region was counted. The results showed that the apoptotic rates of the Ad-SnoN group (51.40% ± 4.11%) were significantly higher than the apoptotic rates of the Ad-EGFP group (16.10% ± 3.38%), while the apoptotic rates of the si-SnoN group (21.6% ± 7.07%) were lower than the apoptotic rates of the si-FITC group (48% ± 4.93%) (Figure 4B).

The expression of fibrosis-associated proteins, including collagen I, collagen III, and TIMP-1 protein decreased in the Ad-SnoN group, compared with the control group and the Ad-EGFP group (all *P* < 0.05, Figure 4C), while that of MMP-13 protein showed no significant difference between these groups (both *P* < 0.05). The expression of collagen I, collagen III, and TIMP-1 protein increased and the expression of MMP-13 decreased in the si-SnoN group, compared with the control group and the si-FITC group (all *P* < 0.05), while that of collagen I showed no difference between these groups (both *P* > 0.05). The expression of collagen I was increased in the OE-SnoN+TGF-β1 group compared with the OE-SnoN group and decreased in the TGF-β1 group (Figure 4D).

### Liver immune microenvironment consists of infiltrating immune cells

**Figure 5.**
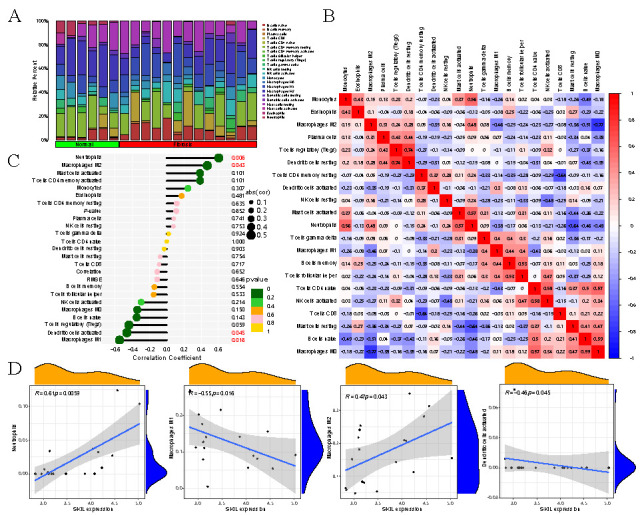
**Liver immune microenvironment consist of infiltrating immune cells**. (A) Correlation matrix results of the immune cells; (B) The correlation of 22 types of immune cells in HF was evaluated. Red indicates positive correlation, while blue indicates negative correlation; (C) Correlation between the *SKIL* gene and infiltrating immune cells in HF; (D) The scatter plots show the correlation between neutrophils, macrophages M1, macrophages M2, and activated dendritic cells with *SKIL* gene expression. HF: Hepatic fibrosis.

In order to evaluate the differences in the immune microenvironment between *SKIL*-downregulated fibrosis tissue and normal tissue, we performed the immune cell infiltration analysis. We used a histogram to represent the composition of 21 immune cells in the sample, and the colors represented the percentage of different immune cells in the sample (Figure 5A). A heat map was then used to assess the correlation between the immune cells (Figure 5B). It is noted that macrophages M2 were positively correlated with plasma cells and neutrophils, while they were negatively correlated with naive B cells and macrophages M0. We then performed the correlation analysis to identify immune cell types associated with the *SKIL* gene (Figure 5C). The results showed that the *SKIL* gene was significantly correlated with neutrophils, macrophages M2, activated dendritic cells, and macrophages M1 (*P* < 0.05). The scatter plots (Figure 5D) show that the *SKIL* gene was positively correlated with neutrophils (*R* ═ 0.61, *P* ═ 0.006) and macrophages M2 (*R* ═ 0.47, *P* ═ 0.04), and negatively correlated with activated dendritic cells (*R* ═ ⎼0.46, *P* ═ 0.05) and macrophages M1 (*R* ═ ⎼0.55, *P* ═ 0.02). These results suggested that the expression of the *SKIL* gene may play an important role in immunity.

### Competitive endogenous RNA network construction

**Figure 6.**
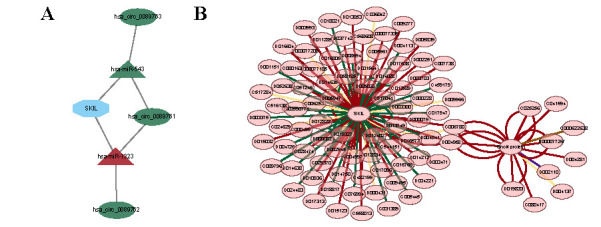
**Construction of ceRNA network and potential drugs network**. (A) The ceRNA regulatory network consists of miRNAs, circRNAs, and *SKIL* mRNA. The triangles represent miRNA, the oval shapes represent circRNA, and the diamond represents mRNA; (B) The gene-chemical compound network. Red: Increase expression; Green: Decrease expression; Yellow: Affect binding; Purple: Decrease phosphorylation; Brown: Increase sumoylation; ceRNA: Competitive endogenous RNA.

To further explore the regulatory mechanism of the *SKIL* gene, we used miRNA as a bridge to establish the relationship between target gene mRNA and circRNA to construct the ceRNA network [17]. First, we found 299 DE-miRNAs in the GSE74872 dataset, including 254 upregulated miRNAs and 45 downregulated miRNAs. 135 DE-circRNAs were found in the GSE134146 dataset, including 62 upregulated circRNAs and 73 downregulated circRNAs. We then took the intersection of DE-miRNAs and miRNAs targeting *SKIL* genes and retained the miRNA–mRNA relationship pairs with opposite expression trends [20]. The intersection of DE-circRNAs and circRNAs targeting the corresponding miRNAs was taken, and circRNA–miRNA relationship pairs with opposite trends were retained [20]. Finally, a ceRNA network with six nodes (1 mRNA, 2 miRNAs, and 3 circRNAs) and six edges was generated (Figure 6A).

### Potential drugs network

To explore the potential drugs for HF, we found 105 drugs targeting *SKIL* and 21 drugs targeting SnoN protein from the Comparative Toxicogenomics Database (CTD). Finally, a gene-chemical compound network containing 128 nodes (*SKIL*, SnoN protein, and 126 chemical compounds) was constructed (Figure 6B).

## Discussion

In the present study, we used a comprehensive bioinformatics method to integrate and analyze multiple datasets. Then, we observed that the expression of *SKIL*/SnoN decreased in the liver of BDL rats and CCL4 rats, whereas the expression of other fibrogenesis-associated proteins increased. Moreover, immunofluorescence and co-IP examinations revealed interactions between SnoN and p-SMAD2 and between SnoN and p-SMAD3 in the cytoplasm of HSC-T6 cells. In addition, we observed that the SnoN overexpression could inhibit the expression of the fibrogenesis-associated proteins, depending on TGF-β1. Furthermore, we revealed that SnoN might be associated with apoptosis and the change of the liver immune microenvironment during HF.

The bioinformatics analysis showed that *SKIL* was downregulated in HF tissues. Our enrichment analysis shows that the enrichment is mainly present in response to extracellular stimuli, response to molecules of bacterial origin, ECM organization, growth factor binding, collagen binding, and TGF-β1 signaling. In the process of HF, macrophages are recruited to the liver to produce cytokines and chemokines, including TGF-β1 and platelet-derived growth factor (PDGF) [21]. TGF-β1 can promote ECM production and inhibit ECM degradation [22]. The analysis of scRNA-seq showed that *SKIL* gene expression was significantly reduced in fibrotic liver tissue samples in HSCs and hepatocytes. Normally, HSCs are silent in liver tissues [23]. When hepatocytes are damaged, HSCs are stimulated by various factors, such as TGF-β1 and PDGF, to synthesize a large amount of ECM and promote the progression of HF. Our results showed that SnoN protein expression was reduced in HSCs activation and the process of HF. In the following experiment verification, we first established the BDL rats’ model and established the CCL4 rats’ model to simulate the process of liver damage during HF. Then we observed that the expression of SnoN decreased in the BDL rats’ liver tissue, while other fibrogenesis-associated proteins increased.

The role of TGF-β1 in the pathological mechanism of HF has been extensively studied, and it is considered to be one of the most critical cytokines to induce HF [24]. Previous studies have shown that SnoN could disrupt the interaction of the phosphorylated region of p-SMAD2/3 with the comediator SMAD4 by forming a wedge between the complexes [25]. TGF-β1 signaling is critical for processes including liver wound repair and ECM production [26]. In our present study, we observed the expression of the co-localization of SnoN and p-SMAD2 as well as SnoN and p-SMAD3 in the cytoplasm of HSC-T6 cells. Furthermore, we identified the interaction between SnoN and p-SMAD2 and p-SMAD3, respectively, by co-IP. Collectively, our study suggests that the way SnoN regulates TGF-β1 signaling in the liver is by binding to phosphorylated SMAD2/3 and disrupting the interaction between p-SMAD2/3.

The fibrotic remodeling of liver tissue is a progressive process which is mainly mediated by the activation of HSCs and the production of ECM [4]. In our present study, the expression of collagen I, collagen III, and TIMP-1 increased in the Ad-SnoN group, indicating that the overexpression of SnoN downregulated the expression of these proteins, which are closely related to the formation of ECM. Our data also showed that the expression of collagen III and TIMP-1 increased, while MMP-13 decreased in the si-SnoN group, indicating the upregulation of fibrosis-associated proteins and downregulation of MMP-13 in HSC-T6 cells with SnoN gene knockdown. The expression of collagen I in the OE-SnoN + TGF-β1 group was intermediate between the OE-SnoN group and the TGF-β1 group, indicating that the inhibitory effect of overexpression of SnoN on collagen I was dependent on TGF-β1.

Our previous study showed that the expression of SnoN protein decreased in hepatic cirrhosis patients with severe HF [27]. In our present study, we found increased expression of TGF-β1 protein as well as TIMP-1, collagen I, and collagen III, which have been proven to be associated with fibrosis formation after liver injury [28, 29]. Some studies have demonstrated that SnoN could inhibit TGF-β1/SMAD signaling pathway in the cytoplasm and the nucleus and strictly regulate the activity of TGF-β1 signaling [7, 25]. SnoN has been studied for its inhibitory function of TGF-β1 signaling in renal tubular tissue, lung tissue, and cancer cells [9, 10, 30]. The expression of SnoN protein decreased in the BDL surgery rats and TGF-β1 stimulated cells, suggesting that SnoN is associated with the process of fibrogenesis in liver. Collagen I and collagen III are the most abundant collagen proteins of the human body, occurring in scar tissue and are directly involved in the pathological process of HF. As a member of the TIMP family, TIMP-1 is a natural inhibitor of matrix metalloproteinases [29]. In addition to its inhibitory role against MMPs, the encoded protein is able to promote cell proliferation in hepatic cells, participate in the migration of HSCs, and have an anti-apoptotic function [31, 32]. As a member of the MMP family, MMP-13 is involved in the breakdown of ECM in the process of HF [33, 34]. The gene expression of SnoN and MMP-13 in the BDL groups showed the tendency to decrease at first and then return to the normal level, which was opposite to the change of ECM-associated genes. The expression of SnoN protein was also associated with collagen III and FN, which could lead to ECM deposition. The inverse correlation between SnoN and TGF-β1 also indicated that SnoN might exert a regulatory function for TGF-β1 signaling and the process of HF.

By comparing the infiltrating immune cells of the fibrotic liver tissue samples and the normal tissue samples, we found that *SKIL*/SnoN-related liver immune microenvironment is considerable. The correlation analysis showed that the *SKIL* gene was positively correlated with neutrophils and macrophages M2 and negatively correlated with activated dendritic cells and macrophages M1. It is known that chronic inflammation is a hallmark of fibrotic disease [35], and the Kupffer cell-derived macrophages have emerged as the key cells in the fibrosis process [36]. Studies have shown that *MYD88* in macrophages enhances HF by activating the nucleotide oligomerization domain (NOD)-like pyrin domain containing protein 3 (NLRP3) inflammasome in HSCs [37]. Macrophages can also promote HF by inducing autophagy in HSCs through the PGE2/EP4 pathway [38]. Therefore, targeting macrophages is a therapeutic approach for HF, but it still needs to be supported by further studies.

The level of SnoN protein plays a major role in the progression of liver fibrosis. Since changes in mRNA levels can also have some effect on protein expression, we constructed the ceRNA regulatory network. In recent years, the ceRNA regulatory network has emerged as a new molecular mechanism affecting miRNA-mediated gene regulation, including miRNAs, mRNAs, and lncRNAs [39]. Among them, miRNA is the core of regulation, which can regulate the expression of target mRNA and can be regulated by lncRNA [17]. In this study, we found two miRNAs related to the *SKIL* gene (miR-543 and miR-1323) and three circRNAs targeting the corresponding miRNAs (circ-0089763, circ-0089761, and circ-0089762). Previous studies have shown that miR-543 acts in cardiac fibrosis [40], renal fibrosis [41], and endometrial fibrosis [42], and miR-1323 promotes tumor progression by targeting *TP53INP1* in hepatocellular carcinoma [43]. However, there is still no study showing that miR-543 and miR-1323 act on HF, which needs to be supported by further studies.

Previous studies have shown that direct inhibition of TGF-β1 to cure HF may lead to many side effects [44, 45]. Hence, a new treatment targeting SnoN may be an effective therapeutic strategy for clinical HF. Drugs targeting the *SKIL* gene and SnoN protein were found in the potential drugs network we constructed. Our previous study showed that the herbal compound 861, a traditional Chinese medical preparation, has protective effects of the hepatic function by decreasing p-SMAD2 and p-SMAD3 protein levels while increasing SnoN protein levels [12]. Together with our previous studies, we have demonstrated the treatment mechanism of herbal compound 861 on HF. Further research is needed to prove that SnoN could serve as a potential target in HF therapy.

## Conclusion

We identified *SKIL* as a DEG associated with HF and demonstrated that the expression of *SKIL/*SnoN was downregulated in fibrotic liver tissue by experiment verification. In addition, SnoN could attenuate the collagen-synthesis via TGF-β1/SMADs signaling pathway, and it could be associated with the liver immune microenvironment.
